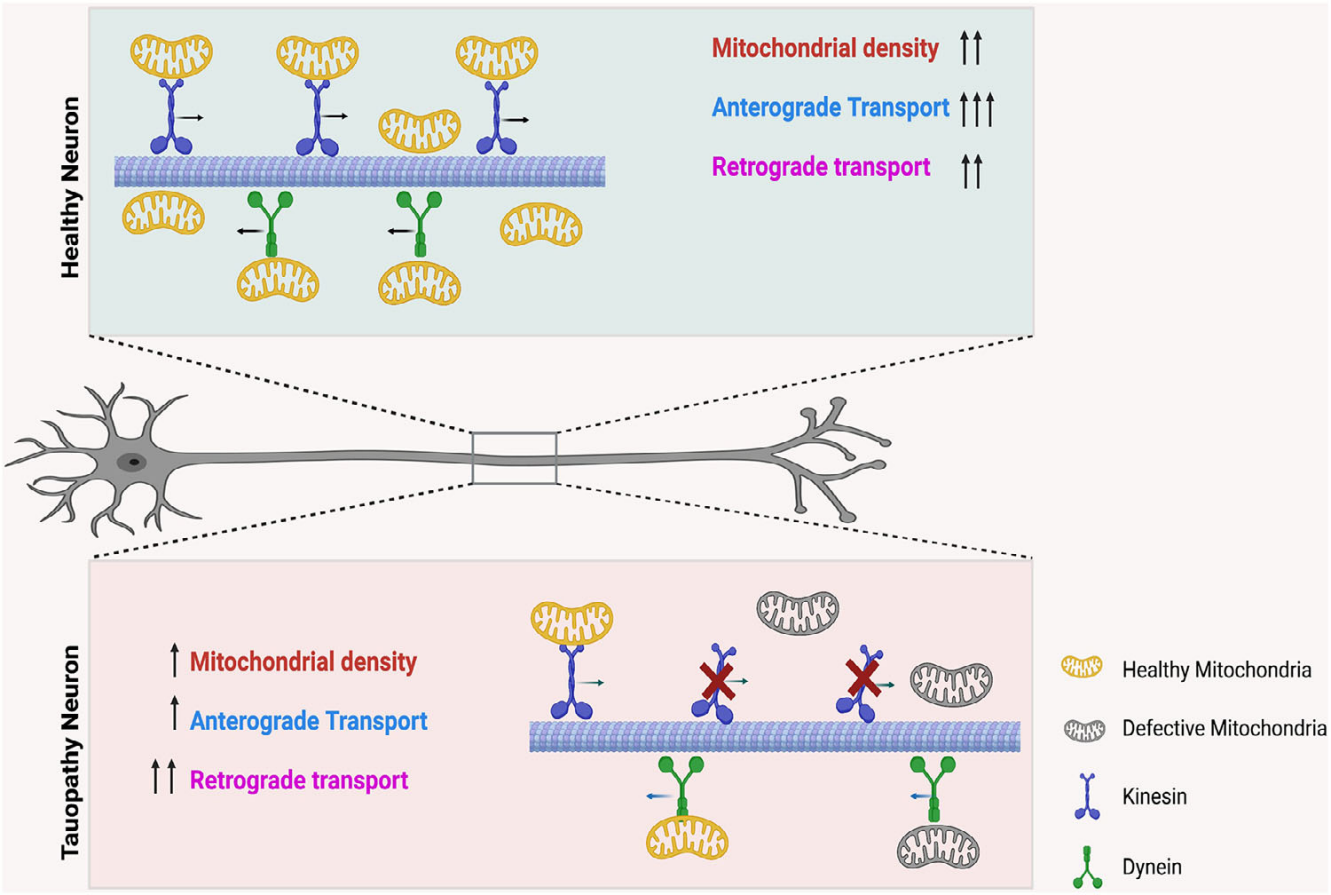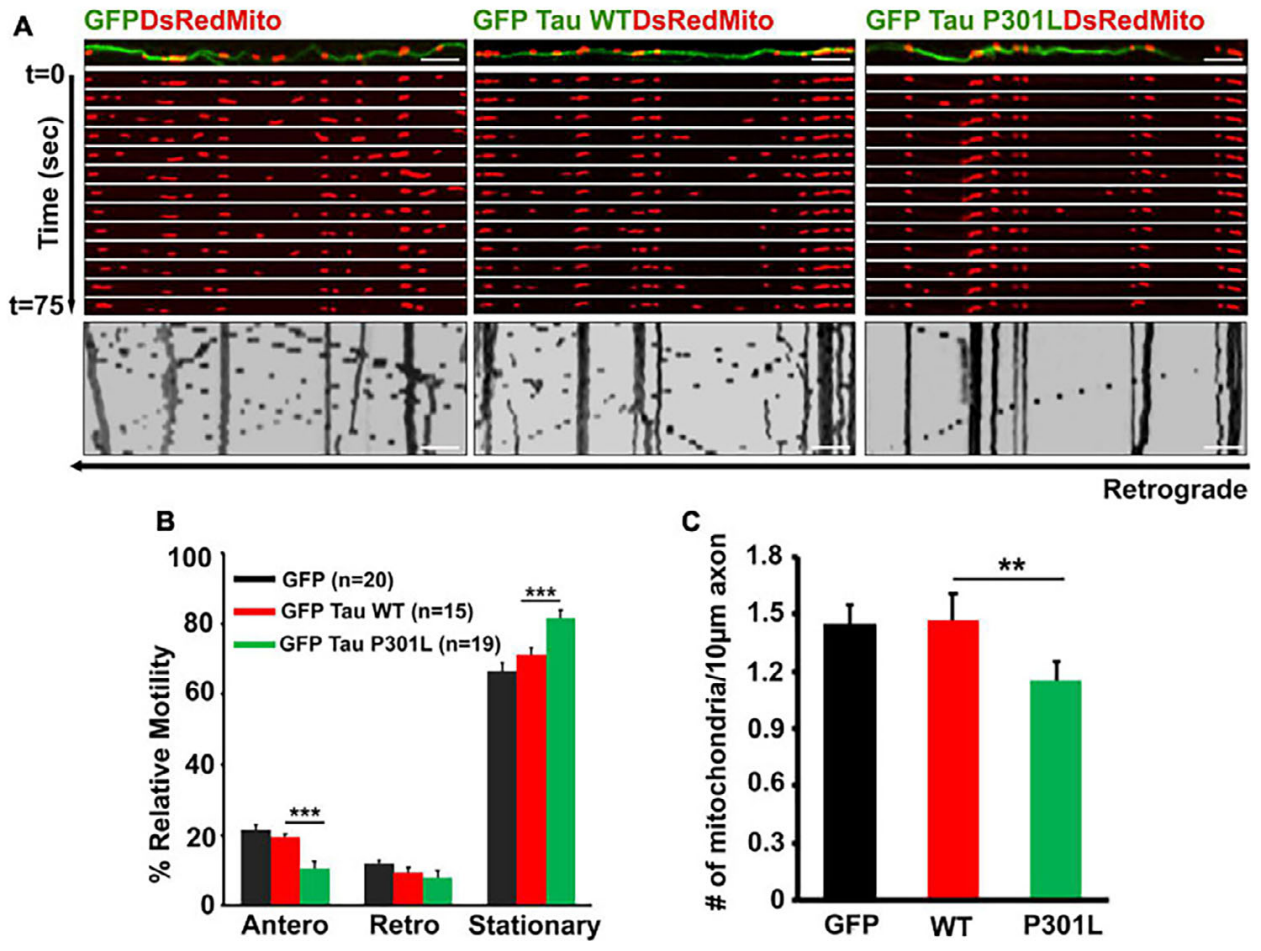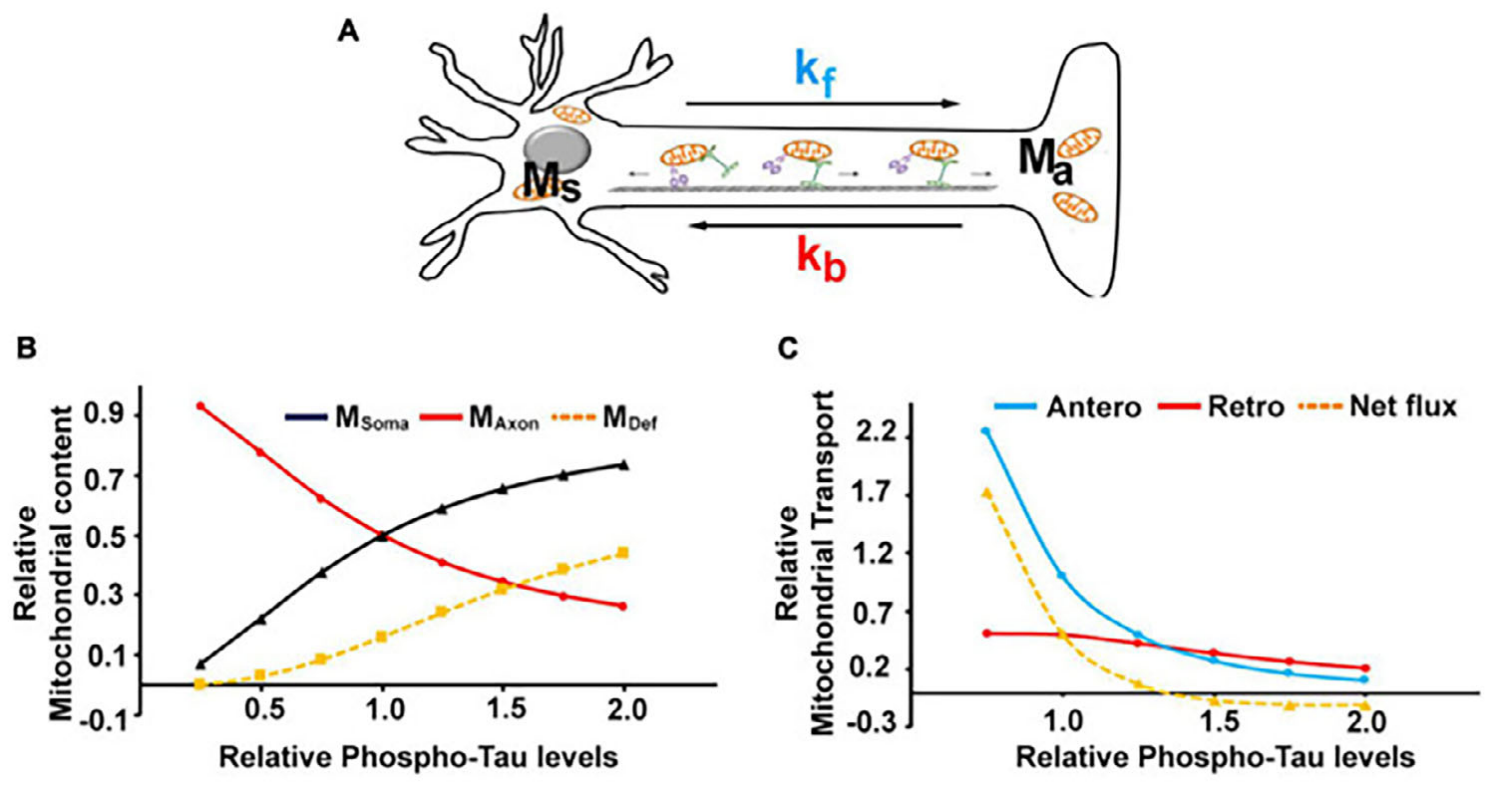# Mitochondrial transport and axonal mitochondrial density in tauopathy neurons

**DOI:** 10.1002/alz70855_100949

**Published:** 2025-12-26

**Authors:** Anusruti Sabui, Prasad Tammineni

**Affiliations:** ^1^ UNIVERSITY OF HYDERABAD, HYDERABAD, TELANGANA, India

## Abstract

**Background:**

Mitochondria are essential organelle for neuronal homeostasis. They supply ATP and buffer calcium at synaptic terminals. The complex structural geometry of neurons poses a unique challenge in transporting mitochondria to synaptic terminals. Anterograde mitochondrial transport is driven by kinesin motors whereas retrograde transport is driven by cytoplasmic dynein. Despite the importance of presynaptic mitochondria, how and whether axonal mitochondrial transport and distribution are altered in tauopathy neurons remain poorly studied.

**Method:**

We studied mitochondrial transport and distribution in neurons expressing the tauopathy‐associated P301L mutant protein. Quantitative analyses of mitochondrial motility and abundance were performed using live‐cell imaging and biochemical assays. We also investigated the interaction between mitochondria and motor proteins, alongside mathematical modeling to evaluate motor activity changes.

**Result:**

P301L neurons exhibited a significant reduction in anterograde mitochondrial transport, with no change observed in retrograde transport. This led to decreased axonal mitochondrial abundance in P301L neurons. Biochemical analyses revealed a reduction in mitochondrial association with kinesin in P301L cells. Interestingly, mathematical modelling of transport dynamics suggested a compensatory increase in dynein activity to maintain retrograde flux.

**Conclusion:**

Our findings demonstrate that decreased kinesin‐mediated anterograde transport coupled with sustained retrograde transport might reduce axonal mitochondrial density in tauopathy neurons. This imbalance may contribute to synaptic deficits observed in Alzheimer's disease and other tauopathies.